# (±)-Catechin—A Mass-Spectrometry-Based Exploration Coordination Complex Formation with Fe^II^ and Fe^III^

**DOI:** 10.3390/cells11060958

**Published:** 2022-03-11

**Authors:** Lenka Kubicova, Gert Bachmann, Wolfram Weckwerth, Vladimir Chobot

**Affiliations:** 1 Division of Molecular Systems Biology, Department of Functional and Evolutionary Ecology, Faculty of Life Sciences, University of Vienna, Djerassiplatz 1, A-1030 Vienna, Austria; lenka.kubicova@univie.ac.at (L.K.); gert.bachmann@univie.ac.at (G.B.); wolfram.weckwerth@univie.ac.at (W.W.); 2 Vienna Metabolomics Center (VIME), University of Vienna, Djerassiplatz 1, A-1030 Vienna, Austria

**Keywords:** Alzheimer’s disease, antioxidant, brine shrimp, Fenton reaction, flavonoid, hydroxyl radical, iron chelate, neurodegeneration, Parkinson’s disease, reactive oxygen species

## Abstract

Catechin is an extensively investigated plant flavan-3-ol with a beneficial impact on human health that is often associated with antioxidant activities and iron coordination complex formation. The aim of this study was to explore these properties with Fe^II^ and Fe^III^ using a combination of nanoelectrospray-mass spectrometry, differential pulse voltammetry, site-specific deoxyribose degradation assay, Fe^II^ autoxidation assay, and brine shrimp mortality assay. Catechin primarily favored coordination complex formation with Fe ions of the stoichiometry catechin:Fe in the ratio of 1:1 or 2:1. In the detected Fe–catechin coordination complexes, Fe^II^ prevailed. Differential pulse voltammetry, the site-specific deoxyribose degradation, and Fe^II^ autoxidation assays proved that coordination complex formation affected catechin’s antioxidant effects. In situ formed Fe–catechin coordination complexes showed no toxic activities in the brine shrimp mortality assay. In summary, catechin has properties for the possible treatment of pathological processes associated with ageing and degeneration, such as Alzheimer’s and Parkinson’s diseases.

## 1. Introduction

Mass spectrometry has been used for many decades as an effective tool for investigating inorganic and organic compounds. Despite being a traditional physico-chemical method, mass spectrometry has recently been used in the development of various modern methodologies in biology, such as proteomics [1] and metabolomics [2,3]. Additionally, mass spectrometry, when combined with electroanalytical or other analytical methods, can provide valuable information about chemical properties of substances that can participate in various cellular physiological processes [4,5,6]. This combination of methods has appeared helpful in formulating hypotheses in cases of hormesis, redox homeodynamic equilibrium (homeostasis), and other biological phenomena [4,5,7]. Electrospray mass spectrometry has contributed to the stoichiometry clarification of transition metal coordination complexes in which natural polyphenols serve as ligands [8,9,10,11].

Iron is an important element that can participate in many physiological processes in organisms [12,13] and probably played a crucial role in the emergence of life [14]. In physiological conditions, iron cellular concentrations are carefully controlled, and iron ions are liganded with various storage molecules, such as ferritin or transferrin [15,16]. However, poorly liganded iron can cause increased oxidative stress in tissues or ferroptosis through the production of reactive oxygen species (ROS), primarily cytotoxic hydroxyl radicals that are generated in the iron-catalyzed Fenton reaction [15,16,17]. Though ROS participate in cellular signal cascades [18], higher ROS concentrations can contribute to cell death and tissue destruction [19]. Neurodegenerative diseases are often associated with the oxidative stress and accumulated poorly liganded iron in brain tissues [15,16,20,21]. Recently, Kletetschka et al. postulated a hypothesis that accumulated Fe particles can cause oscillating magnetic domains in the brain of patients suffering from Alzheimer’s or Parkinson’s disease [22]. Therefore, one of the currently investigated treatment possibilities focuses on antioxidants and other substances that form stable coordination complexes (chelates) with iron ions [23,24,25,26].

In this context, plant polyphenols, especially flavonoids, have attracted attention for several decades due their beneficial effects on human health [27] that also include apparent neuroprotective activities [28,29,30,31]. In clinical praxes, a semisynthetic flavonoid derivative, troxerutin, is used for treatment of various chronic diseases [32]. Assumedly, flavonoids can affect different pathological processes via ROS-scavenging properties and abilities to form coordination complexes with poorly liganded iron [28,33].

**Figure 1:**
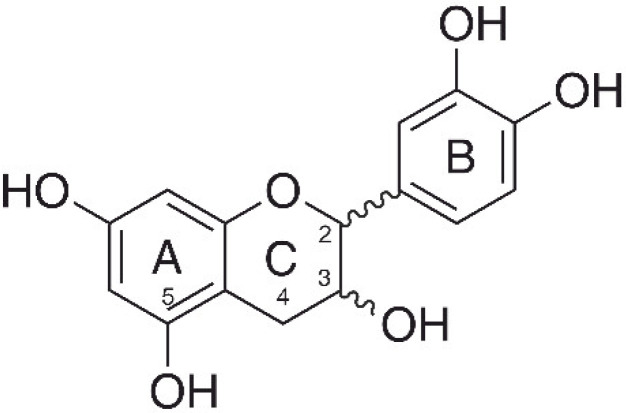
Chemical structure of catechin.

Catechin (Figure 1), a flavan-3-ol, is an extensively investigated flavonoid that commonly occurs in many popular beverages and foods, such as green tea, cocoa, dark chocolate, apples, grapes, and others [34]. Due to its antioxidant properties, catechin shows anti-inflammatory [35,36], and cardio- [35,37] and neuroprotective [35,38] effects. Controversially, catechin was also investigated for possible allelopathic activities. In this context, the researchers claimed that catechin—exuded by the roots of the invasive plant species *Centaurea stoerbe* L.—generated oxidative stress in root tissues of competing plants [39]. However, a detailed redox chemical study provided no evidence for the proposed mode of action [40]. To add to the complexity of effect assessment, the coordination complexes of test compounds can show different redox properties and bioactivities compared to the free test substances [41,42,43].

Fe–catechin coordination complexes have been investigated repeatedly [10,44,45,46]. These studies suggest that the coordination complexes of catechin are primarily formed with the *o*-dihydroxy group of ring B. Catechin lacks a 4-keto group that, together with the 5-hydroxy group, may serve as a second coordination center [33,46]. However, the effects of coordination complex formation on pro- or antioxidant abilities of iron and catechin are not yet fully known. Therefore, we investigated stoichiometry, redox, and toxic properties of Fe–catechin formed in situ coordination complexes. For this purpose, we used nano-ESI–MS (nanoelectrospray ionization–mass spectrometry), differential pulse voltammetry (DPV), deoxyribose degradation assay, Fe^II^ autoxidation assay, and brine shrimp mortality assay. Nano-ESI–MS provides information about stoichiometry of coordination complexes and oxidation stages of central atoms [6]. The DPV and the deoxyribose degradation and Fe^II^ autoxidation assays characterize redox activity changes of the central atoms and ligands [5,6,7]. The brine shrimp mortality assay is often used for toxicological investigations [47,48,49]. In this assay, we explored possible damage to living cells that is caused by Fe overload. It can be caused by increased Fe bioavailability affected by Fe–catechin coordination complexes [50,51]. In our previous investigations, this combination of methods proved very efficient [5,6,7].

## 2. Materials and Methods

### 2.1. Chemicals

(±)-Catechin (catechin throughout the text) and all other used chemicals were purchased from Sigma-Aldrich (Schnelldorf, Germany). Water was of Milli-Q quality (Milli-Q Advantage A10 System, Millipore SAS, Molsheim, France).

### 2.2. Mass Spectrometry Analyses

MS analyses were performed on a Thermo Electron LTQ-Orbitrap XL mass spectrometer equipped with a nanoelectrospray ion source (ThermoFisher Scientific, Bremen, Germany) and operated under Xcalibur software (2.2 version number), in positive ionization mode. The instrument was calibrated using the manufacturer’s calibration standards. The Fourier-transformed, full-scan mass spectra were acquired at a target value of 106 ions with a resolution of 100,000 in the *m*/*z* range of 80–2000; the lock mass option was enabled. Cyclomethicone N5 ions generated in the electrospray process from ambient air (*m*/*z* 371.101230) were used for internal recalibration in real time. This allowed mass accuracies of <1 ppm. Spray voltage was set to 1.8 kV, capillary voltage was 45 V, tube lens offset 150 V, and capillary temperature was set at 180 °C; no sheath gas and auxiliary gas used. Catechin coordination complexes were measured according to Sarowar et al. [11], although the concentration of metals we used was five times lower.

The samples for the nano-ESI–MS were prepared from a 1 mM stock solution of catechin in degassed methanol, adding degassed aqueous FeCl_2_ or FeCl_3_ solution (500 µM) in (catechin:Fe) molar ratios 1:2, 1:1, 2:1, 3:1, and 4:1. Samples were diluted 1:10 or 1:100 with a water/methanol mixture (50:50, *v*:*v*). For the nano-ESI–MS measurement, gold-coated glass emitters (DNU-MS GbR, Berlin, Germany) with 5 µL of this final sample were used. Theoretical masses and characteristic iron isotopic patterns were calculated by Xcalibur version 2.2 (ThermoFisher Scientific, Bremen, Germany).

### 2.3. Differential Pulse Voltammetry

Voltammetric curves were recorded as described previously in detail [6].

The samples were prepared according to the flowing procedures: FeSO_4_ was dissolved in degassed water at a concentration of 10 mM. The stock solution of catechin was prepared in a degassed buffer (0.1 M phosphate buffer at pH 7.4). The ionic strength of the buffer was 1 M adjusted by K_2_SO_4_. The samples for the electrochemical measurements of catechin were prepared by mixing 1 mL of their stock solution with 8 mL of the aqueous buffer solution and 1 mL of water. The final concentration of the catechin was 1 mM. The samples for the coordination complex analysis were prepared by mixing 1 mL of aqueous FeSO_4_ solution with 9 mL of the degassed buffer or buffered catechin solution. Aqueous FeSO_4_ solution was added 2 min before voltammetric curve recording. The final applied molar concentration ratio of catechin:Fe^II^ was 1:2, 1:1 and 2:1. The solutions of electrolytes were degassed by argon for 10 min and measurements were carried out under argon atmosphere at room temperature. The used scan potentials ranged from −600 to 1300 mV.

### 2.4. Deoxyribose Degradation Assay

The procedures of deoxyribose degradation assay were performed as described in detail elsewhere [52]. The used buffer was aqueous solutions of KH_2_PO_4_/KOH (30 mM, pH 7.4) or KH_2_PO_4_/H_3_PO_4_ (30 mM, pH 6.0).

### 2.5. Fe^II^ Autoxidation Assay

The procedures and reaction mechanisms were published by Chobot et al. [34]. The aqueous solutions of KH_2_PO_4_/KOH (30 mM, pH 7.4) or KH_2_PO_4_/H_3_PO_4_ (30 mM, pH 6.0) were used as buffer.

### 2.6. Brine Shrimp Mortality Assay

The procedures were published previously [7]. For each experiment, 0.5 g of *Artemia salina* cysts (NovoTemia, JBL GmbH &Co.KG, Neuhofen, Germany) was allowed to hatch in 25 mL of buffered saline aqueous solution (pH 7.4). The experiment was carried out in aqueous solution of buffered saline (g per 1 L: 8.0 NaCl, 0.2 KH_2_PO_4_, 1.15 Na_2_HPO_4_, 0.2 KCl).

## 3. Results

### 3.1. Mass Spectrometry

Mass spectra proved that catechin forms coordination complexes with both Fe^II^ and Fe^III^ separately (Table 1 and Table 2). The isotopic pattern of the detected complexes corresponded to the characteristic isotopic pattern of iron (^54^Fe 5.8%, ^56^Fe 91.7%, ^57^Fe 2.2%, and ^58^Fe 0.3%).

**Figure 2:**
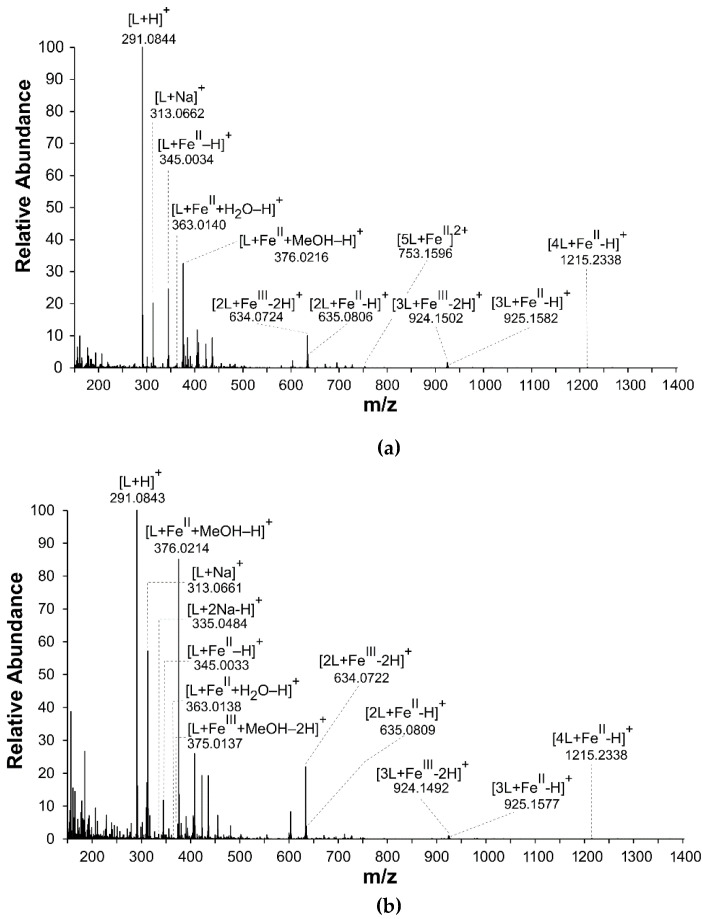
Mass spectra of coordination complexes in solutions of (±)-catechin (L) with (**a**) Fe^II^ and (**b**) Fe^III^, detected by nano-ESI–MS, positive ionization mode. The solutions were prepared by mixing a (±)-catechin solution with Fe^II^ or Fe^III^ solutions in a molar metal-to-ligand ratio of 1:2.

The spectra recorded after addition of Fe^II^ or Fe^III^ to the catechin solution were similar (Figure 2a,b). This suggested that both solution mixtures contained the signals of catechin coordination complexes in which iron occurred as the central atom in both iron oxidation stages. In both experimental setups, the dominant stoichiometry of catechin:Fe was 1:1. In the solutions, coordination complexes with a stoichiometry of catechin:Fe 2:1, 3:1, 4:1 and 5:1 were also detected. Binuclear coordination complexes were not detected.

**Figure 3:**
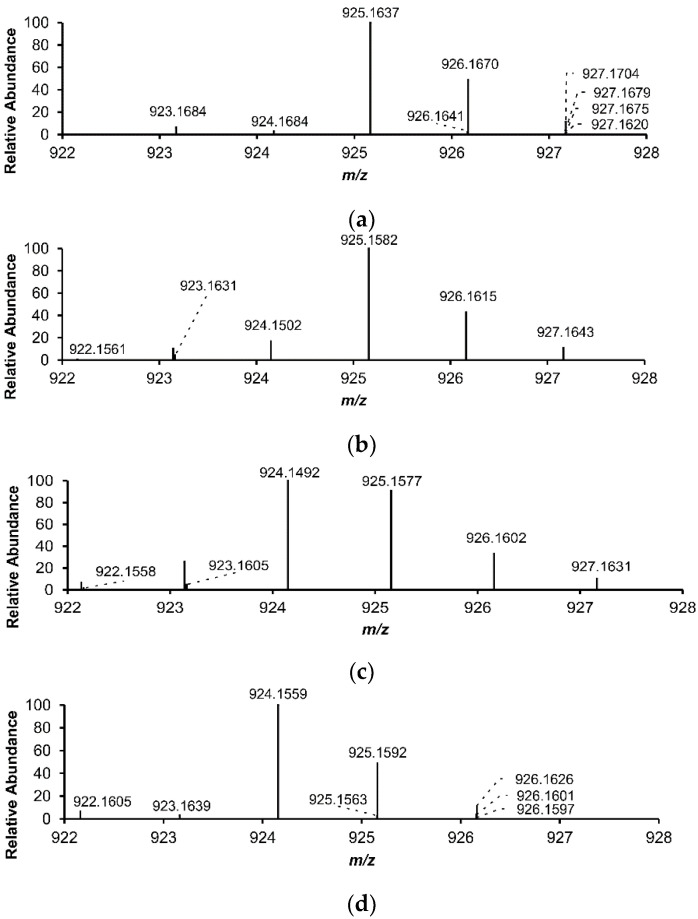
Details of recorded and simulated mass spectra of [3L+Fe^II^-H]^+^ and [3L+Fe^III^-2H]^+^: (**a**) simulated spectrum of (±)-catechin with Fe^II^; (**b**) recorded spectrum of (±)-catechin with Fe^II^ mixture solution; (**c**) recorded spectrum of (±)-catechin with Fe^III^ mixture solution; (**d**) simulated spectrum of (±)-catechin with Fe^III^, detected by nano-ESI–MS, positive ionization mode. The solutions were prepared by mixing the (±)-catechin solution with Fe^II^ or Fe^III^ in a molar metal-to-ligand ratio of 1:2.

A section of the mass spectrum is demonstrated in Figure 3, which proves the existing Fe^II^/Fe^III^ equilibria in the Fe–catechin solutions. The simulated spectra (Figure 3a,d) corresponded to those recorded by pointing out the characteristic Fe isotopic patterns and the presence of both formal iron oxidation stages in the coordination complexes with catechin in the recorded spectra (Figure 3b,c). The peaks of closely similar *m*/*z* values are not distinguished in the measured spectra.

### 3.2. Differential Pulse Voltammetry

**Figure 4:**
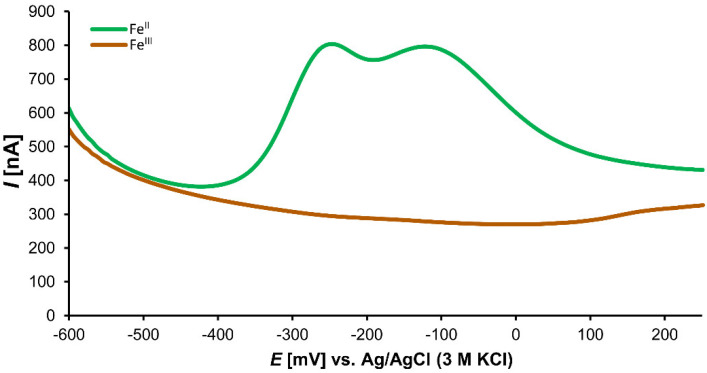
Differential pulse voltammogram of 1 mM solutions of Fe^II^ and Fe^III^ in phosphate buffer (pH 7.4).

The voltammogram of the Fe^II^ solution showed one broad prominent peak with two maxima at −252 and −282 mV (peaks 1a and 1b, respectively, in Figure 4). The formation of coordination complexes of Fe ions with the buffer components explains this phenomenon. Due to the strong ability of Fe^III^ to form many coordination complexes with phosphate that became undetectable, a voltammetric investigation of Fe^III^ solutions with catechin was impossible.

**Figure 5:**
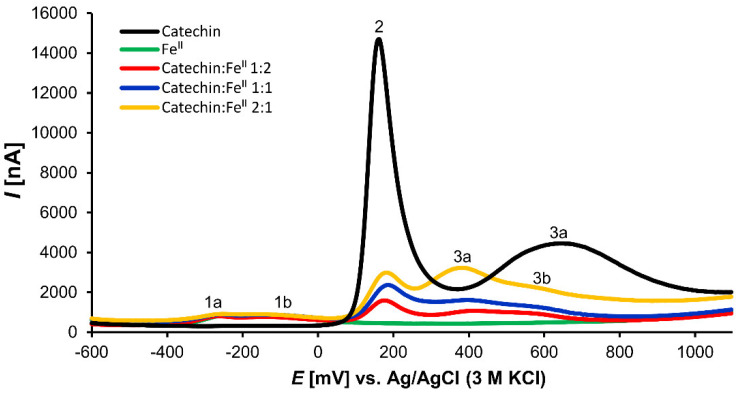
Differential pulse voltammograms of (±)-catechin, Fe^II^, and (±)-catechin:Fe^II^ mixture solutions in phosphate buffer (pH 7.4).

In the solutions of Fe^II^ with catechin, two prominent peaks, 2 (163 mV) and 3a (650 mV), of the catechin voltammetric curve (Figure 5) probably corresponded to the redox reactions of the *o*-dihydroxy group of ring B and the *m*-dihydroxy group of ring A, respectively [53]. Alternatively, Janeiro and Oliveira-Brett proposed that the peak 3a ca be caused by oxidation of the 3-hydroxy group of ring C [54]. After the addition of Fe^II^ solution, the voltammogram curves of catechin dramatically changed (Figure 5). Peak 2 became smaller and peak 3 showed one maximum and shoulder (peaks 3a and 3b). The electrode potentials of 3a and 3b varied according to the ligand:Fe^II^ ratio. With increasing concentration of catechin in the analyzed solution mixture, the electrode redox potential of the peak 3a shifted to the cathodic direction (Table 3).

### 3.3. Deoxyribose Degradation Assay

The deoxyribose degradation assay is used for assessing the pro- or antioxidant effects of tested substances. The oxidant agent is a hydroxyl radical (^•^OH) that is produced by the iron catalyzed Fenton-like reaction (Reaction 1). The ^•^OH radicals attack 2-deoxyribose, which is oxidatively degraded to TBARS [52,55].

Fe^II^ + H_2_O_2_ → Fe^III^ + –OH + ^•^OH (1)

The traditional reaction mixture contains hydrogen peroxide, ascorbic acid, and Fe^III^, which we added as FeCl_3_ (the site-specific arrangement of this assay). In the site-specific arrangement, an amount of Fe ions coordinates 2-deoxyribose. Nevertheless, in this complex, the Fe ions can still catalyze the Fenton-like reaction [55].

The presence of hydrogen peroxide simulates a situation of high oxidative stress in the tissue. After a reduction of Fe^III^ to Fe^II^ by ascorbic acid, the Fenton-like reaction begins. The tested compounds can inhibit 2-deoxyribose degradation either by direct hydroxyl radical scavenging or by affecting Fe catalytic properties if Fe ions are bound in coordination complexes. When hydrogen peroxide and/or ascorbic acid are omitted, these assay variants offer different information.

**Figure 6:**
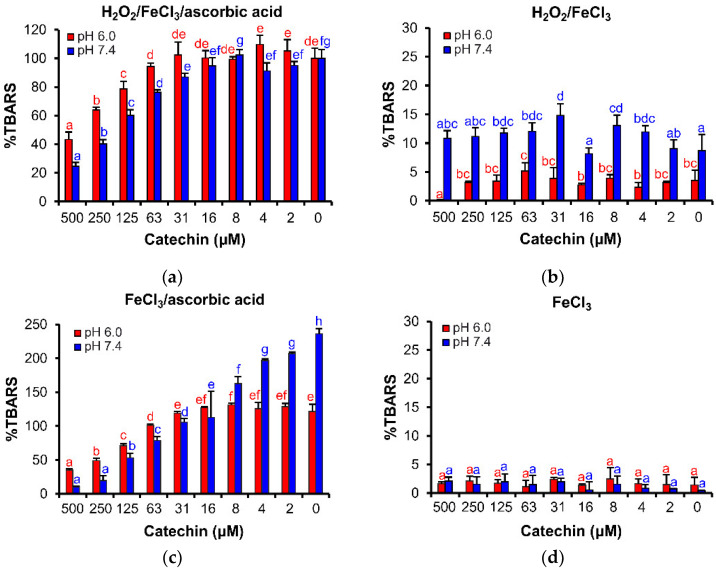
Inhibition effects of (±)-catechin on TBARS formation in the site-specific deoxyribose degradation assay: (**a**) H_2_O_2_/FeCl_3_/ascorbic acid, (**b**) H_2_O_2_/FeCl_3_, (**c**) FeCl_3_/ascorbic acid, and (**d**) FeCl_3_. The bars represent the mean of three replicates (± SD). Letters above the bars indicate significance levels (ANOVA with 95% Duncan’s post hoc test). TBARS: thiobarbituric acid reactive species, SD: standard deviation.

Catechin showed evident antioxidant effects in the H_2_O_2_/FeCl_3_/ascorbic acid and FeCl_3_/ascorbic acid variants (Figure 6a,c). Notably, the antioxidant effects were stronger in pH 7.4 than in pH 6.0. In the H_2_O_2_/FeCl_3_/ascorbic acid variant, catechin inhibited TBARS production in the concentration range of 31–500 µM in the weakly alkaline pH. In the acidic pH of this reaction mixture, the antioxidant effect of catechin appeared in the concentration range of 63–500 µM.

In the FeCl_3_/ascorbic acid variant (Figure 6c), catechin effectively decreased TBARS concentration in pH 7.4 (2–500 µM). In the acidic reaction milieu, catechin was significantly less effective; the antioxidant activity appeared in concentrations higher than 63 µM. The strong difference was probably caused by different dissociations of catechin phenolic groups and partial hydrolysis of the Fe–catechin coordination complexes in the acidic reaction mixture [33,54].

In the other variants, H_2_O_2_/FeCl_3_ and FeCl_3_ (Figure 6b,d), catechin demonstrated no activity because catechin was not able to promote Fe^II^/Fe^III^ redox cycling [52]. Catechin showed no significant pro-oxidant activities in any of the deoxyribose degradation assay variants presented in this article.

### 3.4. Fe^II^ Autoxidation Assay

In this assay, Fe^II^ reduces molecular oxygen to a superoxide anion radical (Reaction 2).

Fe^II^ + O_2_ → Fe^III^ + O_2_^•−^ (2)

Superoxide anion radicals (O_2_^•^^−^) dismutate to hydrogen peroxide (H_2_O_2_), which enters in the Fenton-like reaction with another Fe^II^ and produces hydroxyl radicals (^•^OH). The test compound can decrease ROS concentrations by coordination complex formation with iron ions or by ROS scavenging.

**Figure 7:**
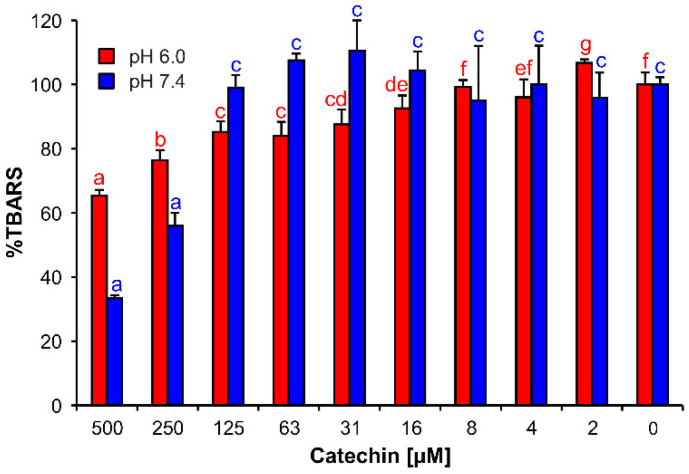
(±)-Catechin effects on TBARS production in Fe^II^ autoxidation assay. The bars are the means of three replications (±SD). Letters above the bars indicate significance levels (ANOVA with 95% Duncan’s post hoc test). TBARS: thiobarbituric acid reactive species, SD: standard deviation.

Catechin showed antioxidant effects in acidic and weakly alkaline pH (Figure 7). Nevertheless, the activities became evident only at the highest concentrations tested, 250 and 500 µM, and only showed in pH 7.4. In pH 6.0, the catechin’s effects were visible in the concentration range 16–500 µM, but less pronounced than in pH 7.4.

### 3.5. Brine Shrimp Mortality Assay

**Figure 8:**
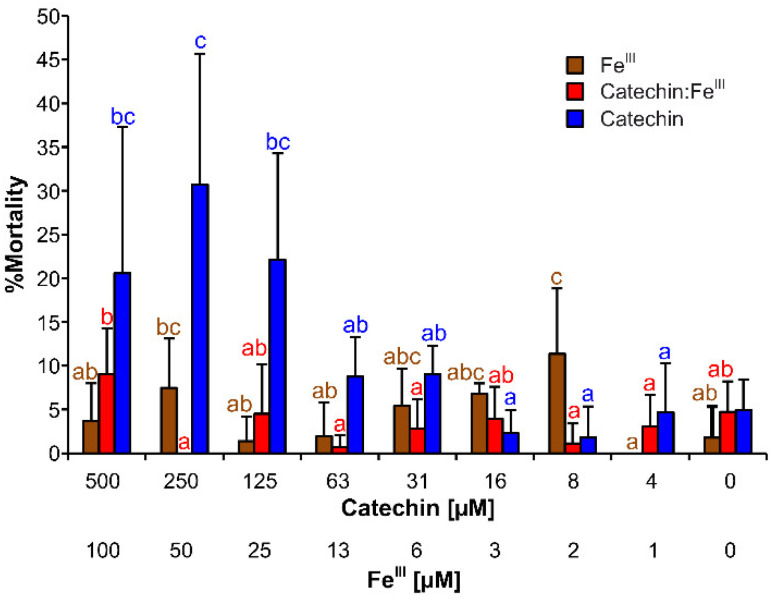
Brine shrimp (*Artemia salina* L.) mortality caused by (±)-catechin in noncoordinated form and as Fe–catechin complexes. The upper scale designates the concentration of (±)-catechin, the lower one designates the concentration of Fe^III^ in the tested solution. The bars represent means of eight replicates (±SD). The different significance levels were determined by Duncan’s post hoc test (95%) and are indicated by the letters a–e above the error bars.

No toxicity of Fe–catechin coordination complexes and FeCl_3_ solution was detectable within the tested concentration range (Figure 8). In the control group, which was treated with catechin solution, mortality increased with catechin concentrations starting from 125 µM. However, average mortality was no more than 31% higher than that of the control.

## 4. Discussion

The strict regulation of ROS and iron concentrations is a crucial process in cells. Therefore, living organisms evolved an array of enzymatic and nonenzymatic mechanisms for the ROS concentration control, including endogenous and exogenous low-molecular-weight antioxidant substances [56,57]. The aim of these antioxidant protection mechanisms is to keep redox homeodynamic equilibrium (homeostasis) but not to scavenge all ROS, which would be detrimental to the cell.

Catechin can stabilize the redox homeodynamic equilibrium by direct reduction of ROS to water or by the formation of Fe–catechin coordination complexes in which the iron is less catalytically active. However, many studies on the abilities of flavonoids to be ligands in iron coordination complexes and to reduce Fe^III^ to Fe^II^ are performed primarily with photometric methods that employ ferrozine or other competitive ligands [45,58,59]. The results of such methods can be affected by the preferred oxidation stage of the liganded metal (Guldberg and Waage’s law) [60]. Consequently, one must be aware that such methods offer only preliminary information, though high throughput at low costs make them an attractive option.

Nano-ESI–MS proved that catechin reduced liganded Fe^III^ to Fe^II^ up to the achievable equilibrium, probably because of the higher crystal field stability of the *d^5^* Fe^II^–configuration compared to that of the *d^6^* Fe^III^–configuration in the Fe–catechin semiquinone coordination complexes. However, this reduction of Fe^III^ to Fe^II^ occurs primarily in acidic pH reaction conditions.

The detailed investigations of redox reactions between Fe and polyphenolic ligands proved that Fe^III^ reduction is slower at higher pH, especially if Fe^III^ coordinates more than one polyphenolic ligand [33]. Additionally, in the phenolic coordination complexes, Fe^II^ is autoxidized by dissolved molecular oxygen to Fe^III^ [33,61]. These counteracting reactions then shape the final equilibria between Fe^II^ and Fe^III^ oxidation stages [61,62]. Moreover, this Fe redox cycling may contribute to the pro-oxidant effects of some polyphenols [33,61,63].

Nano-ESI–MS or spectrophotometric methods have been used for stoichiometry investigations of Fe–catechin coordination complexes. However, various authors reported controversial results. Grzesik et al. reported a stoichiometry of 3:1 for (+)-catechin: Fe^II^ coordination complexes [64]. By contrast, results that were obtained in this study concur with those reported by Mira et al., who identified the most intensive mass spectrometry signals as catechin:Fe 1:1 and 2:1. Similarly, the authors observed the reduction of Fe^III^ to Fe^II^ by flavonoids [44]. In contrast to the investigations of other authors, who observed the Fe reduction in acidic milieu [33,44], we detected an Fe reduction in the catechin coordination complexes in nearly neutral pH.

Electroanalytical methods are efficient tools for redox, antioxidant, and coordination complexes explorations [7,65,66,67]. The DPV concurs with the results of nano-ESI–MS. Peak 2, which probably corresponds to the oxidation of the *o*-dihydroxy groups of ring B, is smaller compared to that of noncoordinated catechin. Furthermore, peak 3a, a possible oxidation of the *m*-dihydroxy group of ring A, shifted the electrode redox potential to the cathodic direction and peak 3b appeared (Figure 5). The cathodic shift of peak 3a supports the notion that the Fe–catechin coordination complexes can be more easily oxidized compared to noncoordinated catechin. Porfirio et al. investigated Fe^II^–flavonoid mixtures, including catechin, by cyclic voltammetry under comparable experimental conditions. These authors assumed that Fe^II^ is liganded by the *o*-dihydroxy groups of ring B due to decreased corresponding oxidation peak in voltammogram [46]. However, we also observed cathodic shifts of the peak corresponding with redox reaction of the *m*-dihydroxy group of ring A. These shifts depended on the catechin:Fe^II^ ratio. Therefore, we cannot exclude any participation of the *m*-dihydroxy group of ring A in coordination complexes formations. As demonstrated by the nano-ESI–MS results (Table 1 and Table 2), the cathodic shift observed in the catechin:Fe differential pulse voltammograms (Table 3) can explain prevalent Fe^II^ in the Fe–catechin coordination complexes. In the primarily occurring coordination complexes of Fe and catechin, catechin proves a more efficient reducing agent.

The stabilization of Fe oxidation stages in the Fe–catechin coordination complexes proved by nano-ESI–MS correspond well with the evident antioxidant effects due to the Fe^II^/Fe^III^ redox cycling of the liganded Fe. In the site-specific deoxyribose assay variants, catechin significantly inhibited thiobarbituric acid reactive species formation, reaction products generated by the oxidation of 2-deoxyribose molecules caused by ^•^OH radicals. Since catechin’s electrode redox potential depends on pH [54], catechin’s antioxidant effect was more apparent at pH 7.4 than at the weakly acidic pH 6.0. Nevertheless, in other reaction conditions of a non-site-specific assay arrangement, when Fe^III^ is added in the form of coordination complex with ethylenediaminetetraacetic acid (EDTA), catechin also showed weak pro-oxidant effects, likely due to relatively low redox potential of the *m*-dihydroxy group of catechin’s ring A [68].

The deoxyribose assay variants with hydrogen peroxide simulate high oxidative stress conditions in cells, for example in damaged mitochondria. In previous studies, catechin protected tissues with rotenone- [69] or 1-methyl-4-phenyl-1,2,3,6-tetrahydropyridine (MPTP)- [70] damaged mitochondria. These substances can harm mitochondrial functions and increase oxidative stress in the cell, especially as shown in nervous tissues [71]. Therefore, they are often used for experimental induction of neurodegenerative processes associated with Parkinson’s disease. Catechin showed a similar protective effect in plants that grew in higher stress conditions [40].

Ascorbic acid is an efficient reduction agent [72] and can accumulate in the human brain up to concentrations of 1–2.6 mM [73]. Additionally, ascorbic acid promotes Fe redox cycling, which leads to molecular oxygen reduction and initialization of the Fenton reaction [72,74].

In the Fe^II^ autoxidation assay, catechin decreased TBARS concentrations in both tested pHs. An inhibition of Fe redox cycling in iron coordination complexes with catechin may have caused this effect. De Sousa et al. and Porfirio et al. reported increased antioxidant activities of metal–flavonoid coordination complexes compared to noncoordinated flavonoids [41,46]. Furthermore, Mahal et al. postulated that Fe^II^–polyphenol coordination complexes may show a superoxide-dismutase-like activity [75]. However, Grzesik et al. did not corroborate any increased antioxidant, superoxide-dismutase-like, or catalase-like activities of Fe^II^–catechin coordination complexes in a comparison to noncoordinated catechin [64].

The formation of coordination complexes can significantly affect, quantitatively or qualitatively, the bioeffects of the central atoms and ligands as well as their bioavailability. For example, the coordination complexes such as cisplatin are used for treatment of malign diseases due to their cytotoxicity [76]; chromium–picolinate coordination complexes are used for a supplementary treatment of diabetes mellitus due to increased chromium bioavailability [77].

The Fe–catechin coordination complexes showed no toxicity to crustacean species *Artemia salina* L., which is sensitive to oxidative stress [49,78,79] and often used for ecotoxicological investigations [80]. Furthermore, the Fe–catechin coordination complexes did not cause oxidative stress or toxic effects due to a possible iron-overload [25,81]. This exploration supports the rationality for adjuvant treatment of iron-overload disease by some iron-chelating agents [81].

Catechin has potential for the treatment of pathological processes associated with ageing and degeneration. However, most flavonoids penetrate poorly into the blood circulation and through the blood–brain barrier. The human plasmatic concentrations of catechin are 0.4–2.2 µM depending on diet [82]. In the brain tissue, the catechin concentration was about 6.2 µmol/100 mg (Wistar rats) [83]. Nevertheless, the polyphenols’ entrance into the brain tissues can be increased by pathological damage to the blood–brain barrier, which is associated with neurodegenerative disease [84]. Therefore, catechin derivatives with improved neuroprotection have recently been synthetized [85,86]. In recent years, the effects of polyphenols on gut-brain axis and gut microorganisms have been discussed [87,88]. In general, it is accepted that gut microbiome modulation is a promising method for preventing neuroinflammations [89]. Nevertheless, the pathological processes involving Fe redox cycling cannot be neglected [23].

## 5. Conclusions

Nano-ESI–MS combined with other methods can offer important insights to interpret catechin’s antioxidant activities in context with coordination complex formation with iron. This study aimed to demonstrate the added value of the inclusion of the method combination in the analysis of redox-active, low-molecular-weight compounds.

With Fe ions, catechin primarily forms coordination complexes of the stoichiometry ligand:Fe 1:1 or 2:1. In these coordination complexes, Fe becomes stabilized mostly as Fe^II^. The coordination complexes with higher numbers of catechin moieties are only formed as minor components in the reaction mixture. Such insights facilitate a better understanding of results than other, more conventionally used experiments yield.

## Figures and Tables

**Table 1:** The main signals of ^56^Fe-catechin coordination complexes in the solutions of (±)-catechin with Fe^II^ analyzed by nano-ESI–MS, positive ionization mode.

Composition	Formula	Intensity %	*m*/*z* Calculated	*m*/*z* Found	Δ (ppm)
[L+H]^+^	[C_15_H_15_O_6_]^+^	100.0	291.0863	291.0844	−1.96
[L+Na]^+^	[C_15_H_14_O_6_Na]^+^	20.2	313.0683	313.0662	−2.01
[L+Fe^II^-H]^+^	[C_15_H_13_O_6_Fe]^+^	24.6	345.0056	345.0034	−2.25
[L+Fe^II^+H_2_O-H]^+^	[C_15_H_15_O_7_Fe]^+^	1.4	363.0162	363.0140	−6.07
[L+Fe^II^+MeOH-H]^+^	[C_16_H_16_O_7_Fe]^+^	32.6	376.0240	376.0216	−6.43
[2L+Fe^III^-2H]^+^	[C_30_H_26_O_12_Fe]^+^	10.1	634.0768	634.0724	−6.89
[2L+Fe^II^-H]^+^	[C_30_H_27_O_12_Fe]^+^	4.3	635.0846	635.0806	−6.42
[3L+Fe^III^-2H]^+^	[C_45_H_40_O_18_Fe]^+^	0.3	924.1559	924.1502	−6.10
[3L+Fe^II^-H]^+^	[C_45_H_41_O_18_Fe]^+^	1.7	925.1637	925.1582	−5.93
[4L+Fe^II^-H]^+^	[C_60_H_55_O_24_Fe]^+^	0.2	1215.2427	1215.2338	−7.34
[5L+Fe^II^]^2+^	[C_75_H_70_O_30_Fe]^2+^	0.5	753.1645	753.1596	−6.51

**Table 2:** The main signals of ^56^Fe-catechin coordination complexes in the solutions of (±)-catechin with Fe^III^ analyzed by nano-ESI–MS, positive ionization mode.

Composition	Formula	Intensity %	*m*/*z* Calculated	*m*/*z* Found	Δ (ppm)
[L+H]^+^	[C_15_H_15_O_6_]^+^	100.0	291.0863	291.0843	−2.03
[L+Na]^+^	[C_15_H_14_O_6_Na]^+^	57.2	313.0683	313.0661	−2.20
[L+2Na-H]^+^	[C_15_H_13_O_6_Na_2_]^+^	1.4	335.0502	335.0484	−1.78
[L+Fe^II^-H]^+^	[C_15_H_13_O_6_Fe]^+^	11.8	345.0056	345.0033	−2.33
[L+Fe^II^+H_2_O-H]^+^	[C_15_H_15_O_7_Fe]^+^	0.3	363.0162	363.0138	−7.17
[L+Fe^III^+MeOH-2H]^+^	[C_16_H_15_O_7_Fe]^+^	3.9	375.0162	375.0137	−6.57
[L+Fe^II^+MeOH-H]^+^	[C_16_H_16_O_7_Fe]^+^	85.1	376.0240	376.0214	−7.03
[2L+Fe^III^-2H]^+^	[C_30_H_26_O_12_Fe]^+^	22.0	634.0768	634.0722	−7.32
[2L+Fe^II^-H]^+^	[C_30_H_27_O_12_Fe]^+^	3.2	635.0846	635.0809	−5.86
[3L+Fe^III^-2H]^+^	[C_45_H_40_O_18_Fe]^+^	1.0	924.1559	924.1492	−7.16
[3L+Fe^II^-H]^+^	[C_45_H_41_O_18_Fe]^+^	0.9	925.1637	925.1577	−6.43
[4L+Fe^II^-H]^+^	[C_60_H_55_O_24_Fe]^+^	0.1	1215.2427	1215.2338	−7.06

**Table 3:** The electrode redox potential of the peak 3a.

Catechin:Fe^II^ Ratio	(mV)
Catechin	650
Catechin:Fe^II^ 1:2	412
Catechin:Fe^II^ 1:1	396
Catechin:Fe^II^ 2:1	381

## Data Availability

Not applicable.
